# Entry of nanoparticles into cells and tissues: status and challenges

**DOI:** 10.3762/bjnano.15.83

**Published:** 2024-08-12

**Authors:** Kirsten Sandvig, Tore Geir Iversen, Tore Skotland

**Affiliations:** 1 Department of Molecular Cell Biology, Institute for Cancer Research, The Norwegian Radium Hospital, Oslo University Hospital, 0379 Oslo, Norway; 2 Centre for Cancer Cell Reprogramming, University of Oslo, 0379 Oslo, Norway; 3 Department of Biosciences, University of Oslo, 0316 Oslo, Norway

**Keywords:** biodegradable, biodistribution, endocytosis, extracellular vesicles, nanomedicine, nanoparticles

## Abstract

In this article we discuss how nanoparticles (NPs) of different compositions may interact with and be internalized by cells, and the consequences of that for cellular functions. A large number of NPs are made with the intention to improve cancer treatment, the goal being to increase the fraction of injected drug delivered to the tumor and thereby improve the therapeutic effect and decrease side effects. Thus, we discuss how NPs are delivered to tumors and some challenges related to investigations of biodistribution, pharmacokinetics, and excretion. Finally, we discuss requirements for bringing NPs into clinical use and aspects when it comes to usage of complex and slowly degraded or nondegradable NPs.

## Introduction

Nanoparticles (NPs) are important tools to diagnose and treat diseases, and have proven useful in basic mechanistic studies of cells and animals. Thus, knowledge about cellular uptake, intracellular transport, and metabolism of NPs in cells, as well as their biodistribution, degradation, and excretion following intravenous (i.v.) injection is required to benefit from NPs as therapeutics or imaging agents in an optimal way. Many different types of NPs have been made; for an overview, see [1]. Doxorubicin encapsulated in liposomes (Doxil^®^/Caelyx^®^) was the first NP-based drug approved for cancer treatment by the US Food and Drug Administration (FDA) in 1995 [2]; this product has a similar therapeutic effect and less side effects than those obtained with the free drug. Later on, also other NPs have been approved for clinical use [1], but there is still a large need for new products. In addition to the development of new types of NPs, there is a knowledge gap when it comes to our understanding of the interaction of NPs with both cells and tissues. However, it is well known that NP properties, such as surface charge, size, and the material they are composed of can affect cellular uptake, biodistribution, and effect on cells.

In the case of NP-based products used for radiotherapy or as imaging agents, it may be sufficient that they end up in the affected areas. Other NPs may, however, need to enter cells in order to deliver the drug to its intracellular target molecules. Also, the cellular location that the NP ends up in may be important to conduct the warranted function. Lipid-based NPs carrying mRNA may have to enter tubular structures in endosomes [3], whereas degradable NPs containing drugs that are not inactivated by low pH or by lysosomal enzymes, may obtain an optimal effect by being transferred to lysosomes. When it comes to entry of NPs into cells, normal tissue, and tumors there are still a number of open questions. Regarding questions on the cellular level: Can one modify the NPs to obtain more efficient targeting and entry? By which endocytic mechanism(s) are the NPs taken up? Will NPs with bound ligands enter by the same mechanism as the free ligands? Will the NPs affect intracellular transport and what are the consequences for the cell or tissue? In vivo, one might want NPs to be transcytosed across a cell layer. However, not much is known about the requirements for NPs to cross a cell layer in this manner when it comes to size, charge, material, and NP-associated ligands. In vivo there are also a number of challenges regarding studies of distribution, half-life, and long-term effects. Furthermore, there is a major challenge in the field of NP research regarding the fact that many articles are being published where the conclusions are not always based on sufficient evidence and knowledge [4]. Thus, there is clearly a need for more cross-disciplinary collaborations.

When we use the terminology NPs here, we mean manufactured NPs which normally are made of only a few different types of molecules. It is now common to include vesicles originating from cells as being NPs. During the last decade there has been an amazing increase in studies of exosomes, small vesicles secreted by fusion of multivesicular bodies (late endosomes) with the plasma membrane of cells. Also, release of other types of vesicles, for instance from the plasma membrane, may play a role in the transfer of information between cells. For a list of various types of extracellular vesicles (EVs), see [5]. For therapeutic purposes, EVs may not only be loaded with drugs after the release from cells, but incubation of cells with drugs may allow drug incorporation into vesicles released by the cells. Recent studies have even suggested that incorporation of drug-containing NPs in cellular membranes might increase the ability of these particles to cross the blood–brain barrier [6]. However, regulatory challenges are high for conventional NPs, and one can easily foresee that demonstrating reproducibility for the production of EVs with their thousands of constituents will be a huge challenge. In this article, we will not cover EVs specifically, but several of the challenges discussed when it comes to uptake studies, and transport in vivo also holds true for these particles. We will start by describing the status and challenges when it comes to cellular uptake mechanisms, and in the last part discuss interactions of NPs with tissues and biodistribution of these particles.

## Endocytic Pathways Involved in Nanoparticle Uptake

### The complexity of endocytosis

**Figure 1:**
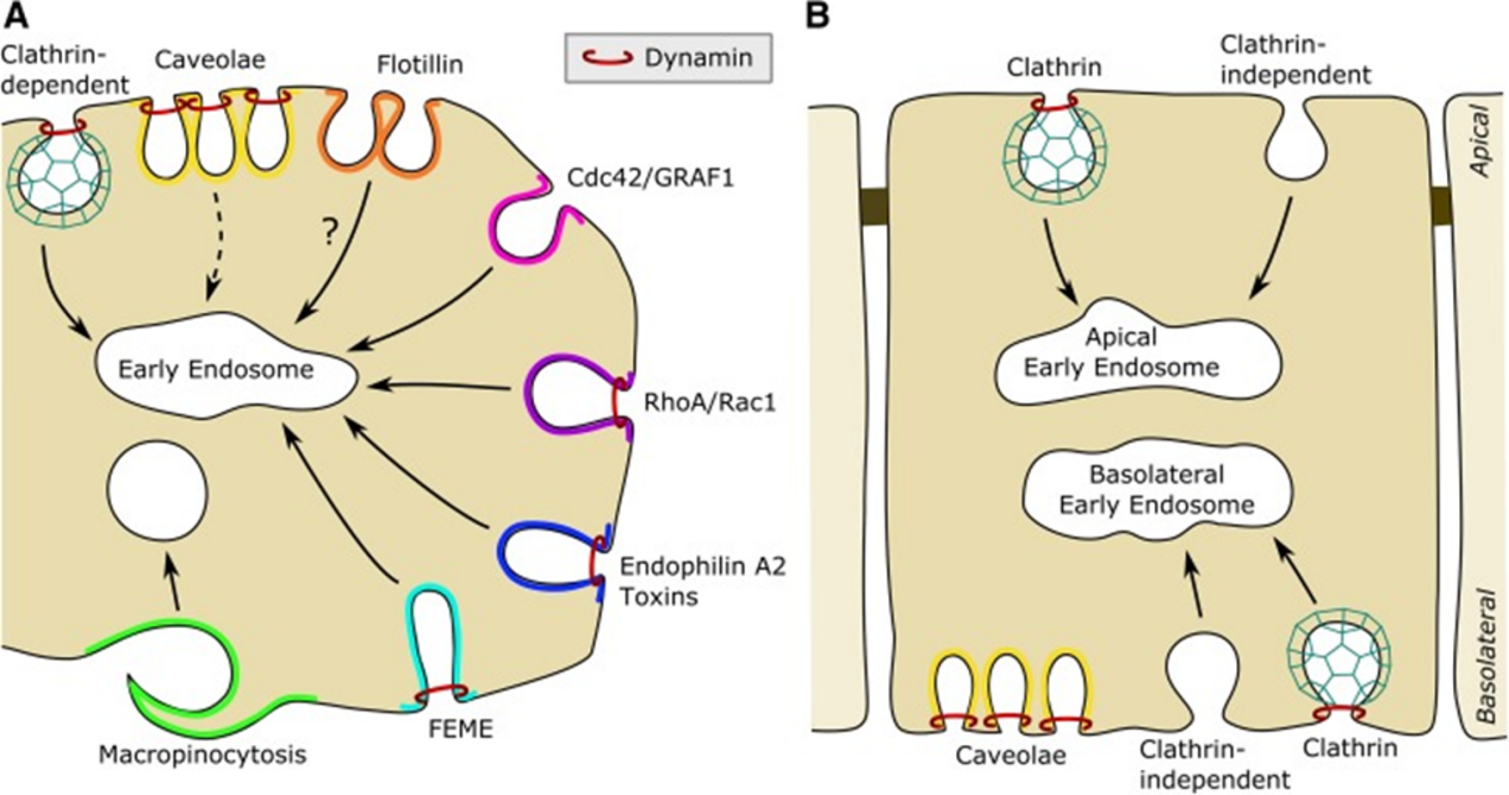
An overview of some endocytic pathways in a non-polarized cell (A) and in a polarized cell (B). Flotillin may contribute to upconcentration of ligands in endocytic invaginations, and dynamin is involved in several endocytic pathways. Caveolae are now regarded as quite stable structures. Note that caveolae are found only at the basolateral side of epithelial cells such as Madin-Darby canine kidney (MDCK) cells, but on both sides of the endothelial cell layer. It should be noted that dynamin was recently reported to stabilize some caveolae [12]. Figure 1 was reproduced from [7] (© 2018 K. Sandvig et al., published by Springer Nature, distributed under the terms of the Creative Commons Attribution 4.0 International License, https://creativecommons.org/licenses/by/4.0).

The field of endocytosis has undergone a remarkable development during the last decades. Today it is clear that there is a variety of uptake mechanisms in cells, and adding to the complexity, they are partially cell-type specific [7–10]. In Figure 1, we have outlined some of the common endocytic pathways in cells. Although we can currently manipulate molecules involved in various pathways in a specific way by siRNA, gene knockout, or CRISPR/Cas, the induced changes can have secondary effects that are not easy to predict. Also, overexpression of proteins, including dominant-negative mutants, may, due to their high concentration, facilitate low-affinity interactions with partner proteins that they normally would not bind to. Furthermore, we have the challenge that a given molecule can be involved in more than one pathway. For instance, cdc42 is involved in macropinocytosis, the CLIC/GEEC (clathrin-independent carrier/glycosylphosphatidylinositol (GPI)-anchored protein-enriched endosomal compartments) pathway, as well as in FEME (fast endophilin-mediated endocytosis) and phagocytosis (an uptake mechanism for large particles mostly found in phagocytes [7–10]). Endophilin is a player when it comes to both clathrin-mediated endocytosis (CME) and FEME, which is an endocytic mechanism induced by growth factors [7–8]. It should be noted that FEME is dependent on the formation of endophilin-positive assemblies on the plasma membrane, and this step in FEME is blocked by a number of inhibitors that target other mechanisms [11]. Importantly, dynamin is required for the formation of vesicles by numerous mechanisms [7–8], but was recently reported to stabilize some caveolae [12]. Furthermore, when interfering with one uptake mechanism, the cell may respond by increasing others [13]. Importantly, knockdown of caveolin may lead to increased uptake via the CLIC/GEEC pathway [14], and a similar phenomenon is seen after knockdown of cavin [15]. To which extent the CLIC/GEEC pathway is important for endocytosis of NPs has not been explored in detail, although it, in some cells, can have a high uptake capacity [16]. The understanding and complexity of these mechanisms have been further increased by the finding that some galectins, such as galectin-3 and galectin-8, can drive cellular uptake by cross-linking glycolipids [17–18]. Interestingly, it was recently published that globular particles with regularly spaced green fluorescent protein (GFP) and a diameter of 40 nm could induce membrane curvature and be internalized when binding to glycosylphosphatidyl-anchored GFP nanobodies [19]. Vesicle formation was energy dependent and dynamin independent, but the details concerning uptake have not yet been published. Thus, whether the molecules are the same ones as those involved in the CLIC/GEEC pathway is not yet known.

The use of inhibitors of endocytic mechanisms to determine how a ligand or NP is internalized is challenging. One can often read that the investigators have used “well-established inhibitors” to investigate the uptake mechanisms involved, but as discussed below, such compounds should be used with caution. One should also keep in mind that a pharmacological inhibitor that affects the uptake of a particle may not necessarily change the formation of an endocytic vesicle, but could change the location and movement of the receptor(s) binding the particle on the plasma membrane [20].

The complexity of endocytosis increases when it comes to polarized cells. For instance, it has been demonstrated that apical and basolateral endocytosis in epithelial cells differ in the sense that caveolae are found only on the basolateral side [21–22]. Interestingly, in polarized epithelial cells such as in an MDCK cell layer, the apical and basolateral uptake is differentially regulated by several signaling pathways, for instance by stimulation of protein kinase A [7]. To which extent this is a general phenomenon is still unknown. In contrast, in endothelial cells caveolae can be seen on both sides of the endothelial cell layer, and caveolae have been reported to be involved in transcytosis across the cell layer [23]. It should, however, be noted that some endothelial cell layers have such a short distance between the poles of the cells that alternative mechanisms for transendothelial transport have been suggested [24]. Also, in non-polarized cells the growth conditions may affect the endocytic pathways and the physiology of the cells. One should be aware that increasing cell density can increase the rate of endocytosis [25–26] and also change the lipid composition of the cells [27–28]. Both the amounts and composition of glycosphingolipids and phospholipids differ in cells grown at high and low density, increasing the chances that also other processes than endocytosis, such as recycling, degradation, and signaling are also regulated by cell density.

**Figure 2:**
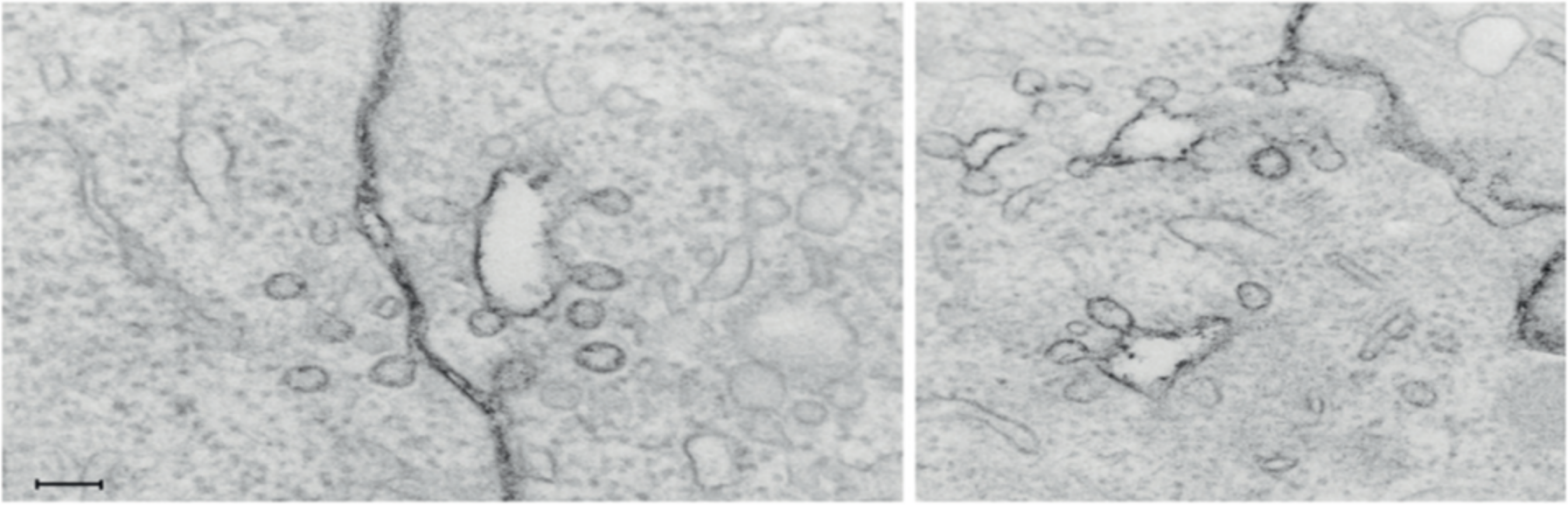
The black staining of the membranes obtained by adding ruthenium red during cell fixation reveals that caveolae, which may appear to be free vesicles in the cytosol, are surface connected. The scale bar is 100 nm. Figure 2 was reprinted from [29], Current Opinion in Cell Biology, vol. 23, by K. Sandvig; S. Pust; T. Skotland; B. van Deurs, “Clathrin-independent endocytosis: mechanisms and function“, pages 413-420, Copyright (2011), with permission from Elsevier. This content is not subject to CC BY 4.0.

**Figure 3:**
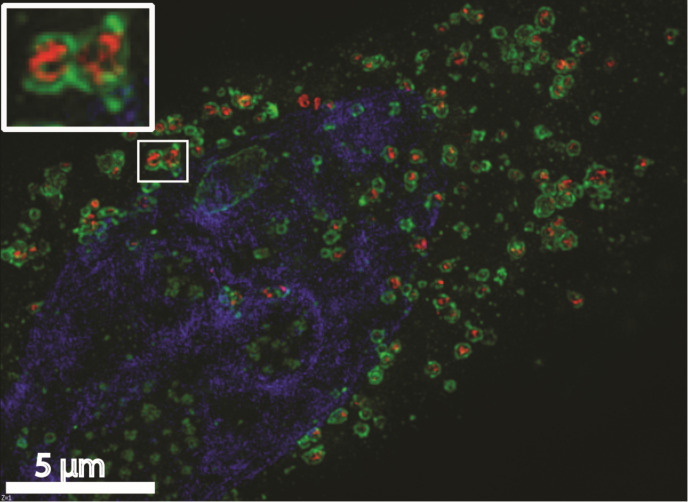
SIM image showing photosensitizer–chitosan conjugate polymeric NPs within lysosomes. MDA-MB-231 cells were incubated with the NPs (red) for 2 h, followed by a washout and chasing for 2 h at 37 °C. Then, the cells were fixed and stained with an antibody against the lysosomal marker LAMP-1 (green); nuclei were stained with DAPI (blue).

In studies of uptake and transport of NPs, it is essential to determine whether the particle is in a sealed vesicle or whether it is still at the cell surface but present in an invagination of the cell. This can be performed by different methods. If electron microscopy (EM) is used, it is important that serial sectioning is performed. Otherwise, one may see a particle which is apparently internalized, since it is far from the cell surface, but it might still be in an invagination. However, even in high-impact articles one can see that conclusions are drawn based on insufficient evidence and thus might be wrong and misleading for the field. Also, addition of ruthenium red during fixation in preparation for EM is helpful to decide if a compound is internalized; see Figure 2 [29]. Other microscopy techniques that are useful for such studies are correlative light and electron microscopy (CLEM) [30], confocal microscopy with Z-stacks [4], and structured illumination microscopy (SIM) which can also demonstrate in which organelles the NPs are localized. The SIM image shown in Figure 3 was obtained using the NPs described in [31]. Another aspect when it comes to studies of endocytosis is the kinetics of the processes. If the number of internalized NPs is measured after a relatively long time (hours), not only the endocytic uptake plays a role for the readout, but also a fraction of the NPs may have been recycled, degraded, or transcytosed. Furthermore, the accumulated particles and their degradation products may start to affect the cells.

### Functions of caveolae, caveolin and cavin

In this section we provide some comments specifically about caveolae, the small caveolin-/cavin-coated structures, which are often reported to be involved in endocytosis of NPs. Caveolae are known to have different functions, one being to provide membrane upon mechanical stress [32]. They can thereby prevent membrane disruption, and reform in an ATP-dependent manner [32]. However, they can clearly pinch off, a process that can be stimulated by a cross-linking ligand such as the simian virus 40 (SV40) [33]. It is known that after being released from the plasma membrane, they may fuse with normal early endocytic vesicles [34]. In 2001, it was published that a separate organelle, the caveosome, was formed, and the internalized ligands could be directly transferred to the endoplasmic reticulum, thereby avoiding ending up in lysosomes and being degraded [33]. However, the same authors published in 2010 that this was wrong, and they advised that the name caveosome should not be used [34]. In spite of this, one can still see authors describing that they aim at getting transport to the caveosome, thereby avoiding lysosomal degradation. Thus, it may take many years to correct opinions formed after publication of erroneous conclusions in high-impact journals.

One should keep in mind that the diameter of caveolae is small, about 60–80 nm, making it unlikely that these structures function in efficient uptake of particles that are much larger. A common tool used to investigate whether caveolae are involved in the uptake of NPs is cyclodextrin, which extracts cholesterol from the plasma membrane and flattens caveolae. However, cholesterol is required for a number of uptake mechanisms (Table 1), for instance for CME and macropinocytosis [35–36]. Most likely cholesterol will turn out to be required for all endocytic pathways. One function of cholesterol in the membrane is to decrease membrane permeability to small ions such as K^+^ and Ca^2+^. The gradients of these ions across the plasma membrane is large; there is much more K^+^ and much less Ca^2+^ in the cytosol than outside the cells. If too much cholesterol is extracted from the plasma membrane, leakage and secondary effects arise. For instance, lowering the K^+^ concentration will decrease protein synthesis, and increasing Ca^2+^ has a number of effects on cellular processes. One can often see that release of lactate dehydrogenase is used to check for leakage, but this molecule is far too large to give a sensitive readout of leakage. Also, statins, known for a long time to be inhibitors of the rate-limiting enzyme in cholesterol synthesis (3-hydroxy-3-methyl-glutaryl-coenzyme A reductase; HMG-CoA reductase), are often used to test if cholesterol is involved in endocytosis of NPs. It should, however, be noted that treatment of cells with statins may not only reduce the total amount of cell cholesterol, but was reported to have major effects on intracellular transport due to aberrant Rab prenylation, caused by reduced formation of geranylgeranyl pyrophosphate, a downstream product of HMG-CoA reductase [37]. This example illustrates that much is still to be learnt about intracellular transport, and that one has to be careful when interpreting data obtained in the presence of inhibitors.

**Table 1:** Inhibitors used to study endocytosis.

Agent	Effect	Pathways affected	Comments/Pitfalls

dynasore	inhibitor of GTPase activity of dynamin [38]	several; see Figure 1	loose inhibitory activity by binding to serum proteins [38]; not specific for dynamin [39–40]
dyngo	inhibitor of dynamin function; six times more potent than dynasore [41]	as for dynasore	loose inhibitory activity by binding to serum proteins [41]; not specific for dynamin [39–40]
amiloride (or its derivative EIPA)	inhibits Na^+^/H^+^ exchange and lowers cytosolic pH close to the membrane; prevents Rac1 and Cdc42 signaling [42–43] and endophilin-positive assemblies at the plasma membrane [11]	macropinocytosis; FEME [11]	may inhibit amino acid transport [44]
cytochalasin D	inhibits actin polymerization [45]	macropinocytosis and several other endocytic mechanisms	not efficient in all adherent cells [45], except for macropinocytosis; inhibits CME at high membrane tension (see text)
latrunculin A	sequesters actin monomers, blocks actin polymerization, and may thus lead to actin filament disassembly [45]	as for cytochalasin D	as for cytochalasin D
chlorpromazine	relocalization of AP2 and clathrin from the plasma membrane to endosomes [46]	CME [46]	not efficient in all cell lines (see text)
pitstop 2	interferes with binding to the N-terminal domain of clathrin [47]	CME	not specific [48–49] (see text)
genistein	inhibitor of several tyrosine kinases	inhibits caveolae pinching [50]; often used as a caveolae inhibitor, but not specific for this process	affects several processes (see text)
methyl-β-cyclodextrin	cholesterol depletion by extracting cholesterol	macropinocytosis and both CME and clathrin-independent endocytosis; not specific for caveolae (see text)	should be checked for possible leakage of K^+^ (more sensitive than protein leakage)
filipin	interacts with cholesterol [51]	flatten caveolae [52]; affect also other membrane structures	unstable in solution; toxic [51]
nystatin	interacts with cholesterol [51]	structure similar to filipin	toxic [51]
2-deoxy-ᴅ-glucose/sodium azide	inhibition of ATP production	all energy dependent pathways	tolerated by cells for limited time

Another compound which is misused as a “specific” inhibitor of caveolae-mediated uptake is genistein, which is a general inhibitor of tyrosine phosphorylation, and which therefore will prevent for instance CME of the epidermal growth factor (EGF) receptor [53] and growth-factor-induced ruffling [54]. One should be aware that recruitment of caveolin to the plasma membrane not necessarily can be used as evidence for a subsequent uptake via caveolae. In fact, there are flat caveolin-coated areas at the plasma membrane where caveolin seems to prevent endocytic uptake of cholera toxin and autocrine mobility factor (AMF) [55]. Caveolin clearly has effects not related to caveolae [56], and the same is the case for its partner cavin [57].

Another misunderstanding that can lead to erroneous conclusions is that if a ligand becomes colocalized with cholera toxin in a vesicle, this can be used as proof for uptake from caveolae. In fact, cholera toxin can cause transmembrane signaling and may induce pinching off of caveolae itself, and cholera toxin has been shown to be internalized by different endocytic mechanisms [58–60]. Uptake via caveolae may be a minor route; introduction of caveolae in Caco-2 cells by transfection with caveolin did not increase the uptake of cholera toxin [58].

### Macropinocytosis and nanoparticle uptake

Several of the NPs studied are too large to fit into vesicles originating from most of the mechanisms shown in Figure 1. However, as described for the uptake of several types of bacteria, macropinocytosis seems to be involved in the uptake of NPs. When not binding to the cell surface, NPs can be taken up by macropinocytosis. However, if binding to the cell surface and cross-linking plasma membrane lipids or proteins, they may even induce their own uptake [61–62]. For instance, cross-linking of receptors by quantum dots (QDs) with Tat proteins can induce Rac activation and macropinocytosis [61]. Similarly, cross-linking caused by the galactose-binding toxin ricin bound to QDs also changes the uptake mechanism of ricin [62]. Thus, the characteristics for multivalent binding of ligands bound to particles can be very different from that of the ligand itself. In the case of ricin, it was shown that the macropinocytosis inhibitor EIPA (5-(*N*-ethyl-*N*-isopropyl)-amiloride) which inhibits the Na^+^/H^+^ exchanger, inhibited uptake of ricin bound to QDs, whereas this was not the case for ricin as such. The uptake of ricin bound to QDs was also inhibited by the expression of dominant-negative dynamin. In this context, macropinocytosis originating from circular ruffles has been reported to be dynamin dependent [63–64]. Thus, there are reasons to expect that when targeting a particle to the cell surface, the resulting uptake will be dependent on the density of the ligand on the particle and the particle diameter; these factors may induce cross-linking to a different extent. It is well known that binding of external ligands to transmembrane receptors can induce conformational changes in the receptor and result in signaling. It is perhaps not common knowledge that not only toxin-induced, but also antibody-induced cross-linking of glycolipids, such as Gb3, the receptor for Shiga toxin, or GM1, the receptor for cholera toxin, can induce transmembrane signaling and changes in intracellular transport and organelles; for a review, see [7]. Thus, NPs targeting glycolipids may cause similar changes.

For therapeutic purposes, it might be an advantage if a given NP with a drug that is supposed to kill the target cell induces macropinocytosis and thereby increases drug uptake. However, increased uptake of nutrients by macropinocytosis has been shown to increase the survival/growth of cancer cells [65–66]. Thus, if one does not succeed in killing the cell, one may end up with stimulating cancer cell growth, and not at all with the intended outcome.

### Clathrin-mediated endocytosis and nanoparticle uptake

Clathrin-mediated endocytosis can function as an efficient uptake mechanism for relatively small molecules and NPs. The diameter of clathrin-coated invaginations is about 100 nm, and it has a turnover of about 1 min; for a review, see [67]. A commonly used tool to interfere with this process is chlorpromazine, which affects clathrin in some cell types but not in others [68]. When trying to block CME one may use transferrin as a control to see that the treatment reduces the uptake of this ligand. Chlorpromazine has been reported to lead to relocalization of clathrin and the adaptor AP2 from the plasma membrane and to endosomes [46]. Notably, this is associated with an inhibition of the recycling of receptors for α_2_-macroglobulin, EGF, and transferrin, and a concomitant depletion of these receptors at the plasma membrane [46]. One might expect that other receptors that normally recycle after uptake from clathrin-coated pits are also affected in a similar manner, and that NPs targeting such receptors might have a reduced binding to the cell surface after chlorpromazine treatment. Furthermore, ruffling and macropinocytosis stimulated by such growth factors might therefore also be reduced, but this has to our knowledge not yet been investigated. Importantly, the dependency of CME on actin has been found to depend on membrane tension [69]. Thus, any treatment that affects cell morphology and thus may change membrane tension, could have an effect on actin dependency. In agreement with this is the finding that the insertion of lysolipids into the membrane made transferrin uptake more dependent on actin [70].

Knockdown of a molecule such as clathrin heavy chain can also be used in endocytosis studies. However, since clathrin is also present on endosomes and in the Golgi apparatus, one can, in spite of an apparently specific change, expect a number of secondary changes, such as on recycling, retrograde transport to the Golgi apparatus, and transport from the trans-Golgi area to endosomes. Although different types of CME inhibitors are available, they may not be specific (e.g., pitstop 2 can also inhibit clathrin-independent endocytosis [48–49]). Moreover, one should be aware that the action of some inhibitors of dynamin, such as dynasore and Dyngo, is inhibited by the presence of serum [38,41], and also that these inhibitors are not specific (see Table 1) [39–40].

## Intracellular Effects of Accumulation and Degradation of Nanoparticles

It has been known for years that even small particles such as QDs affect intracellular pathways, even those which are not used by the particles. For instance, small QDs that are not transported to the Golgi apparatus, may still change the extent of Golgi transport of other ligands [71]. Although QDs with transferrin seem to enter in the same way as transferrin, recycling is strongly reduced [71]. The explanation for this is not known; however, it is possible that, although the hydrodynamic diameter of these particles was quite small (50 nm), they could be too large to enter the tubular structures involved in recycling. It would not be surprising if interfering with receptor/membrane transport in various organelles will have secondary effects on membrane transport in general. Also, if not degradable but ending up in endosomes or lysosomes, one would expect that an accumulation of NPs may disturb membrane trafficking and lysosomal degradation in these cells. In line with this, it turns out that the uptake of various NPs changes the release of vesicles and their content from cells. The NP type and the concentration, as well as the cell type studied are important for determining whether there is a decrease or increase in vesicle secretion. Moreover, exposure to various types of NPs (Au, Ag, SiO_2_, and Fe_3_O_4_) was found to change the content of EV-containing miRNAs [72]. It is important to understand the mechanisms involved to analyze exosome markers with and without incubation with NPs.

Nanoparticles under development for drug delivery are made from different types of material, and even NPs with slight differences in chemical composition but having the same size and zeta potential have turned out to have very different effects on cells. They have for instance very different effects on autophagy in a cellular system [73], and it can be difficult to predict cellular/organ effects after delivery in vivo [74]. Similarly, although lipid-encapsulated RNAs have turned out to be very successful in vaccination against Covid-19, the nonendogenous lipids present could have unexpected effects.

## Entry of Nanoparticles into Tissue

### How do nanoparticles enter tumor tissue from blood?

The so-called enhanced permeability and retention (EPR) effect has for many years been stated to be the main mechanism for delivery of NPs from blood into tumors. This effect is explained by the fact that more NPs enter and are retained in tumors due to a leakier endothelial cell layer (enhanced permeability) and less drainage by the lymphatic system (enhanced retention) in tumor tissues [75–76]. A couple of years ago, Sindhwani et al. challenged this view and concluded that most NPs enter tumors by an active transport over the endothelial cell layer [77]. Most of the data published to support such an active transport mechanism was based on comparing data from normal mice with those obtained using the “Zombie” mouse model, in which the whole animal was fixed by transcardiac perfusion with a solution containing formaldehyde and glutaraldehyde. We have discussed earlier several issues we regard as weaknesses in this study, and concluded that more studies are needed to demonstrate if or to which extent active transport over the endothelial cell layer is a major contributor to the transport of NPs from blood into tumors [78].

### Biodistribution, pharmacokinetics and excretion studies

In order to have a nanoparticle-based product approved for clinical use, it is necessary to perform a number of different absorption, distribution, metabolism, and excretion (ADME) studies [79–80]. We discussed a couple of years ago that there are several challenges in how to perform and interpret data obtained during such studies [81]. As described above for in vitro studies, there are many similarities both regarding possibilities and challenges in how to perform such studies with NPs and EVs. Both types of particles can be labelled using partly similar strategies, and the same imaging modalities are used to study biodistribution, pharmacokinetics, and excretion of such particles. Thus, although we here focus on NPs, most of what we discuss could be directly transferred to similar discussions about EVs.

We refer to Table 2 and our previous detailed discussions of the modalities used for whole-body imaging (i.e., positron emission tomography (PET), single-photon emission computed tomography (SPECT), optical/fluorescence, magnetic resonance imaging (MRI), computed tomography (CT; using X-ray) and ultrasound imaging), and the spatial resolution, depth of imaging, sensitivity, and advantages/disadvantages of using various methodological approaches [81]. So far, most ADME studies with small animals have been performed using fluorescence; however, labelling with radioactive isotopes for PET or SPECT imaging is growing in popularity due to enhanced imaging depth and spatial resolution for whole-body imaging. It is often useful to apply a combination of different imaging modalities when performing ADME studies of NPs. It is important to be aware that most methods used to label NPs or EVs with a fluorescence molecule or a radioactive isotope will change their surface properties, and this should be taken into careful consideration during studies; for a thorough discussion see [81].

**Table 2:** Imaging modalities and contrast agents used in the clinic.

Imaging modality	Contrast agents/probes used	Spatial resolution	Limit for depth of imaging	Sensitivity	Amount injected in humans

PET	radiolabel (positron emitters; e.g., ^18^F, ^11^C, ^13^N, ^62^Cu,^68^Ga,^124^I)	1–2 mm	no	10^−11^–10^−12^ M	nanograms
SPECT	radiolabel (gamma emitters; e.g., ^99m^Tc, ^111^In, ^123^I, ^125^I, ^131^I, ^201^Tl)	1–2 mm	no	10^−10^–10^−11^ M	micrograms
optical/ fluorescence^a^	fluorescent molecules	≈1/10 of depth of imaging	from <1 cm and up to 10 cm	10^−9^–10^−11^ M	micrograms to milligrams
MRI	paramagnetic metals (e.g., Gd or Mn) or ferromagnetic particles	25–100 µm	no	10^−3^–10^−5^ M^b^	milligrams to grams
CT	iodine-containing molecules. Other heavy atoms can be used.	50–200 µm	no	10^−2^–10^−3^ M	grams
ultrasound	gas-filled microbubbles	50–500 µm	no?^c^	see footnote^d^	micrograms to milligrams

^a^Large differences in the parameters listed due to a variety of optical methods used. Depth of imaging less than 1 cm for reflectance imaging; up to approx. 10 cm with fluorescence tomographic techniques. ^b^Cells labeled with iron oxide NPs may be detected with a sensitivity close to that of SPECT. ^c^Reduced signals from deep tissues, depending upon the frequency used. ^d^Depends very much on bubble size and structure, and the frequency used; single bubbles may be detected.

Table 2 was adapted from [81] (© 2022 T. Skotland et al., published by Elsevier, distributed under the terms of the Creative Commons Attribution 4.0 International License, https://creativecommons.org/licenses/by/4.0)

Independently of which modality used, one should always ask the following questions: What are we now seeing? Is it the labelled NPs or is it just the label that in some way has been released from the NPs? The same question should, of course, be raised in in vitro studies; however, the challenge may be even bigger for in vivo studies where the experiments are often performed over longer periods. Discussions about how the size and charge of NPs contribute to biodistribution and pharmacokinetics have been ongoing for many years. Such discussions often focus on the properties of attached polyethylene glycol (PEG) chains (e.g., density and chain lengths) and how these chains affect the binding of proteins to the NPs. The protein corona most often contains proteins involved in complement activation, macrophage uptake, lipid metabolism, and blood coagulation [82–85]. A challenge regarding the importance of such studies is that in vitro studies using mice plasma was reported to give a different protein corona than that obtained in vivo in mice [86]. Also, in vitro studies performed in the presence of various serum concentrations revealed different types of protein corona and endocytic uptake [87]. Thus, more studies are required to investigate if and how in vitro protein binding studies can help us to explain the in vivo behavior of NPs.

Following i.v. injection of NPs with a diameter of 5 nm, approximately half of the injected dose can be expected to be rapidly excreted in urine, whereas there will be almost no excretion in urine of NPs with a diameter of 10 nm or larger [88]. Most of the injected NPs accumulate in the liver, whereas the uptake in the spleen may be similar or higher if measuring the uptake per gram of tissue. There are reports indicating that the liver is more efficient than the spleen in taking up NPs up to 200 nm, whereas the spleen may take up more of even larger NPs. As recently discussed, more studies are needed to learn about the contribution of the spleen versus liver for uptake of NPs as well as for understanding the contributions from various types of liver cells (Kupffer cells, i.e., the liver macrophages, liver sinusoidal endothelial cells (LSEC), and hepatocytes) in NP uptake [81]. A very recent study points to the importance of interactions between PEG-NPs with (apo)lipoproteins and scavenger receptors, and postulates that the high presence of these receptors on Kupffer cells and LSEC is responsible for the rapid uptake of PEG-NPs in the liver. The authors concluded that this is more important than macrophage interactions following NP opsonization [89].

One important outcome of ADME studies is to describe not only where in the body most of the particles are taken up, but also to learn if and how fast they are degraded/excreted. It is important to learn about these issues early in the development process of a new product candidate for clinical use. The reason is that it is necessary to perform expensive safety studies for a much longer period for products which are not degraded/excreted or where these processes are very slow [80]. Thus, in order for an NP-based product to be approved for clinical use, it is advantageous to produce NPs that are based on endogenous molecules such as lipids or human albumin compared to nondegradable or very slowly degradable nonendogenous molecules [90]. It should be mentioned that there is not much data showing biodegradation/excretion of NPs. Iron oxide-based NPs have, however, been used as safe contrast agents for MRI for many years and have been shown to be degradable both in solutions in vitro [91] and after injection in rats [92].

In vivo studies are essential to evaluate the efficacy of drug-loaded NPs since it is not only the tumor cells that are affected by treatment but also the microenvironment in the tumor, for instance the macrophages. We showed some years ago that cabazitaxel-loaded NPs had a good therapeutic effect on a human breast cancer xenograft in mice, and discussed if an increased ratio of M1/M2 (anti-tumorigenic/pro-tumorigenic) macrophages was important for the therapeutic effect [93]. We have recently investigated in more detail the changes occurring in tumor-associated myeloid cells in another breast cancer xenograft model and found a strong reduction in immunosuppressive function of macrophages [94]. An influence of NPs on macrophage recruitment, differentiation, and polarization has also been reported by others [95–96]. Thus, a combined effect on the tumor cells and the tumor microenvironment may contribute to a successful treatment.

A new type of lipid-based NP was developed in order to obtain vaccines against the Covid-19 pandemic. These lipid-based products, although showing many similarities to other lipid-based NPs, contain a new type of synthetic ionizable cationic lipids [97] which can facilitate the transport of mRNA from endosomes and into the cytosol. There, it can be translated into peptides/proteins which can serve as antigens for the formation of antibodies against Covid-19 virus proteins. A key question regarding the possibility to benefit from using similar lipid-based products for drug delivery into the cytosol is whether sufficient drug is delivered when not benefitting from the cytosolic amplification as in the case of mRNA. Another issue that needs attention regarding the use of such lipid-based NPs for drug delivery is whether ionizable synthetic lipids (key constituents in these products) may create immune responses; see discussion in [97].

### How much of the injected dose reach the target?

Most studies performed with drug-loaded NPs have been performed with the goal of targeting and treating tumors, and there are numerous reports showing that only a very small amount (most often less than 1%) of the injected NPs ends up in the target [98]. It is important to keep in mind that, although for some NP-based products such as for imaging or radiotherapy, it can be sufficient that the NPs end up in the tumor area. However, for many drug-loaded NPs this may not be good enough, as the drug needs to reach its intracellular target (often in the cytosol) to be active. Thus, quantification of NPs in the tumor area is important to understand what happens with the NPs following i.v. injection; however, far from being sufficient to evaluate the therapeutic potential of such NPs. Furthermore, in most articles describing the amount of drug taken up in the tumor area, it has not been investigated if the drug is still present inside the NPs and not able to reach its target. To our knowledge, there is only one publication describing a method to determine how much of a drug is released from the NPs or is still being encapsulated within the NPs [99]. This method which was used to analyze plasma samples, is both complicated and time-consuming and one should not expect researchers to include such analyses in exploratory (early research) studies.

Many researchers try to increase the fraction of the injected dose ending up in the diseased area by employing targeting molecules to receptors in this area. Studies performed more than 20 years ago using only targeting molecules (e.g. antibodies), with the goal of developing radiotherapeutics or imaging agents, showed that only 0.01% or less of the injected dose was retained per gram of tumor in humans; reviewed in [100]. In mice, one often sees that approx. 5% of the injected dose of NPs without targeting molecules ends up in 1 g of tumor. Thus, it would not be surprising if addition of targeting molecules to NPs does not result in a significant increase in the fraction of the injected dose reaching the tumor in mice. However, such targeting molecules may improve the therapeutic effect of the NPs by helping the drug to reach its specific target. It might help by directing the NPs to the right cell type, it might increase the uptake into the cells, or it could prevent release of the NPs from the affected area. When discussing such issues, it is important to keep in mind that although the authors of many articles have reported that very little of the injected antibody dose is reaching the target, antibody-based products have been a great success during recent years with more than 100 of such products approved by the FDA [101].

## Bringing Nanoparticle-Based Products into Clinical Use

It is a great challenge to obtain approval for use of new products in the clinic. We have shortly discussed above the advantages of producing NPs consisting of endogenous lipids or albumin, or having other substances that are degraded and excreted. Although the so-called quantum dots have been found useful for basic studies of cells and small animals, they are too toxic to be approved for human use [90,102].

It should be noted that although there are many similarities in the challenges regarding what is needed to bring NPs and EVs into clinical use, there are main differences regarding the number of different molecules these particles consist of. Whereas NPs may consist of only a few different molecules, EVs may consist of thousands of various molecules making it a huge challenge to document the reproducibility of EV batches. This is, in our opinion, a very important issue that is often overlooked regarding what is needed to bring such products into clinical use.

For a further discussion about the challenges of bringing new NP-based products into clinical use, see [90]. That article includes descriptions about the risk/benefit evaluations that one can expect pharmaceutical companies to perform before starting the development of new drugs. There is also a discussion about the need for extensive interdisciplinary collaboration between experts in many different scientific disciplines in order to improve the quality of studies and, hopefully, obtain many new NP-based products entering clinical use in the near future. A very recent article provides useful advice about the regulatory landscape (an issue which most people in academia are not very familiar with) and describes possible strategies in order to bring new nanomedicines to the market [103].

## Summary

Future studies of NP uptake into cells and tissues are important to get a complete mechanistic understanding of how they affect cells of different types, how one may modify the NP synthesis, and which drugs to use to maximize the possibilities for successful use in nanomedicine. How stable should the NPs be under different physiological conditions to reach their target and then exert their action? To which extent can we make particles that both stimulate immune reactions to, for instance, cancer cells and, at the same time, are toxic to the cells we want to kill? If the NPs need to be transported across the endothelial cell layer to reach a tumor, does the mechanism vary and can it be optimized? One should remember that by making complex NPs it could be difficult to produce reproducible batches and get approval for clinical use. Clearly there are a number of challenges related to different fields in science and physiology. Thus, for future success, interdisciplinary collaboration is required.

## Data Availability

Data sharing is not applicable as no new data was generated or analyzed in this study.
